# Progressive compressive myelopathy induced by a rare primary isolated thoracic vertebral hydatid cyst: A case report

**DOI:** 10.1097/MD.0000000000025177

**Published:** 2021-03-19

**Authors:** Bei Zhang, Li Zhang, Hongli Zhou, Junwei Tian, Jiping Wang

**Affiliations:** a Department of Radiology, First Hospital of Jilin University; b Pain Department, China-Japan Union Hospital of Jilin University, No. 126; c Department of Bone and Joint Surgery, First Hospital of Jilin University Changchun, Changchun, China.

**Keywords:** echinococcus granulosus, extradural, hydatid cyst, spinal compression, thoracic vertebral column

## Abstract

**Rationale:**

Hydatid cyst is a disease caused by the larvae of Echinococcus spp. The larvae often reside in the liver, lungs, and brain. Occasionally, a primary isolated thoracic vertebral hydatid cyst is reported to cause severe complications. Various diseases may lead to the development of progressive compressive myelopathy. Herein, we report a rare case of a primary isolated thoracic vertebral hydatid cyst with compressive myelopathy.

**Patient concerns:**

A 57-year-old female had numbness and weakness in the lower limbs for a span of 3-months.

**Diagnosis:**

Thoracic magnetic resonance imaging (MRI) showed that an isolated mass was observed in the T5 vertebral body, which compressed the spinal cord. The diagnosis was confirmed after surgical excision, and Echinococcus granulosus was found to be the etiologic factor.

**Interventions:**

The patient underwent laminectomy with no complications.

**Outcomes:**

After surgical decompression, the patient made slow and measurable progress. While relatively rare in the non-pastoral area, the primary isolated thoracic vertebral column hydatid cyst may be considered as a possible etiology of atypical extradural spinal compression.

**Lessons:**

This case illustrates the complexity of spinal echinococcosis manifestations and the necessity of an interdisciplinary approach.

## 1 Introduction

Hydatid disease is a chronic parasitic disease caused by Echinococcus larvae. Hydatid disease is known to be a fatal zoonosis that could endanger human health worldwide, particularly in pastoral areas, semi agricultural, and semi pastoral areas where animal husbandry has been developed. Hydatid cyst is potentially fatal, with a mortality rate from 0.9% to 3.6%.^[1]^

The two primary forms of human infection with Echinococcosis are cystic echinococcosis and alveolar echinococcosis. Cystic echinococcosis is more common, which is known as Echinococcus granulosus. By analyzing the phylogenetic relationship of Echinococcus granulosus, the G1-G3 genotype of Echinococcus granulosus was classified as *E. granulosus* sensu stricto, G4 genotype as *E. equinus*, G5 genotype as *E. ortleppi*, G6-G10 genotype as *E. canadensis*, and *E. granulosus* lion strain as *E. felidis*.^[2,3]^

After human infection with Echinococcus granulosus, one or more echinococci can develop in the body, primarily in the liver and lungs, and often in kidneys, spleen, muscles, central nervous system, and eyes, occasionally in the bones (2% to 5%). We report a case of T-5 vertebral body and T-5-6 level extradural Echinococcus granulosus causing lower-limb paralysis in the absence of clinical history and exposure. The disease has only been diagnosed after histopathological examination. Imaging and clinical manifestations can accumulate more experience and improve the understanding of Echinococcus granulosus vertebral lesions.^[4]^

## 2 Case presentation

A 57-year-old female without a remarkable history was admitted to our hospital on September 8th, 2019. She had a three-month history of numbness and weakness of the lower limbs, severely aggravated by walking. Subsequently, she developed progressive difficulty with ambulation and bladder dysfunction issues.

The general physical examination was found to be normal. The neurological assessment showed grade 0/5 strength of the lower limbs by the Medical Research Council, including a sensory loss below the xiphisternum. The patient is an urban resident in northeast China who works as a civilian in a private company. The patient has been stable in the past and has denied any history of tuberculosis, tumor, diabetes, and surgery. The patient had no experience in living pastoral areas and raising livestock. She has no history of smoking and consuming alcohol. No tumors in the nervous system and autoimmune diseases have been traced in her family.

**Figure 1:**
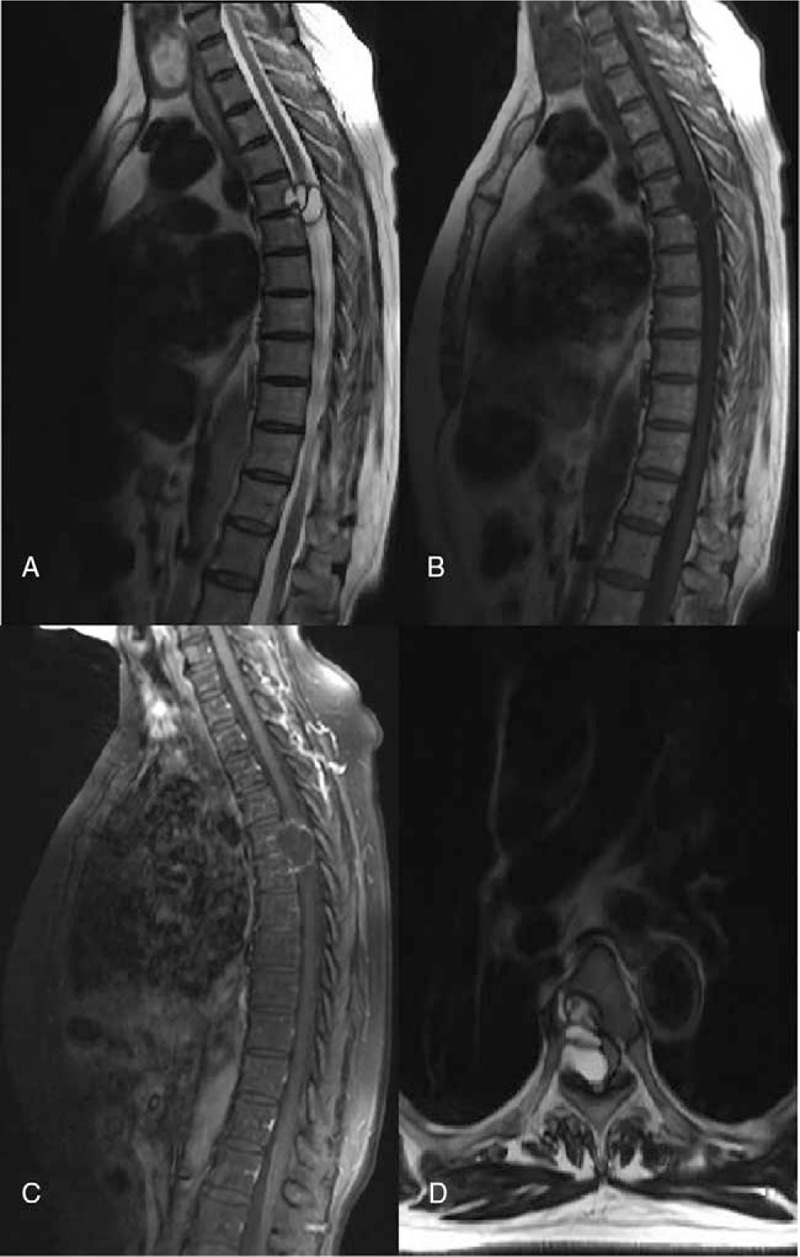
Preoperative MR images. (A and B) Sagittal T2/T1-weighted image showing that a cystic lesion with septations has been embedded in T5; (C) Sagittal enhanced T1-weighted image showing the cystic lesion wall and enhanced septa; (D) Axial T2-weighted image showing compression of the spinal cord by the extradural cystic lesion.

Thoracic magnetic resonance imaging (MRI) results showed that an isolated extradural cystic mass was embedded in the T-5 vertebral body (September 10, 2019). The lesion was hypointense by T-l-weighted imaging and hyperintense by T-2-weighted imaging. Furthermore, the lesion had spread to the spinal canal by compressing the spinal cord from the right side, causing cord compression. Besides, a few thin septa have been found in the cystic lesion. Peripheral and septum enhancement of contrast T-1-weighted imaging was recorded, whereas an abnormal signal has been noticed in the spinal cord (Fig. 1). The cervical MR, lumbar MR, chest CT, brain MRI and abdominal enhanced CT scanning (September 10, 2019) failed to reveal additional lesions. A space-occupying lesion was first considered by combining with the results obtained from thoracic MRI. Laboratory analyses found normal for hemogram, C-reactive protein levels, and the erythrocyte sedimentation rate. The peripheral leukocyte count was found as 7810 per μL, with 1.3% of eosinophils.

Under general anesthesia, exploration, decompression, and pedicle screw fixation were performed for space-occupying lesions in the T-5 and T-6 regions on September 14, 2019. During surgery, the T-5 vertebral body was invaded by a lesion containing cysts of various sizes. There was no extension of the lesion into the rib. The mass, which was exposed after laminectomy, had contained cysts of various sizes embedded in the T-5 vertebral body and found on the right side of the extradural, with adherence to the dura, during which clear fluid was seen inside the lesions, and the wall consisted of white tissue. The completely extradural lesion compressed the dural sac and its contents from an external location. Adequate decompression of the spinal cord was performed, and the mass was peeled away entirely to prevent the rupture of the lesion. The surgical region was washed off using 20% hypertonic saline.

**Figure 2:**
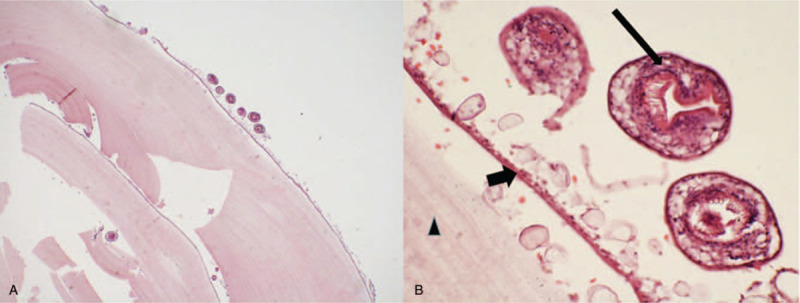
Pathological HE staining results. A shows multiple cystic lesions and fragments of bony trabeculae (by hematoxylin and eosin staining: × 40 magnification); B demonstrates that the laminated layer (triangle sign) in contact with the proliferating membrane (short arrow) from which protoscoleces (long arrow) are detached (hematoxylin and eosin staining: × 400 magnification).

Histopathological (Fig. 2) and parasitological studies supported the diagnosis of echinococcosis granulosus (genotype G1). The results of serum evaluation found an IgG ELISA of 3.20 (norm < 0.9), and an *E. multilocularis* Em2 þ ELISA of 0.56 (norm < 0.9), thus confirming the diagnosis of infection with *E. granulosus*.

**Figure 3:**
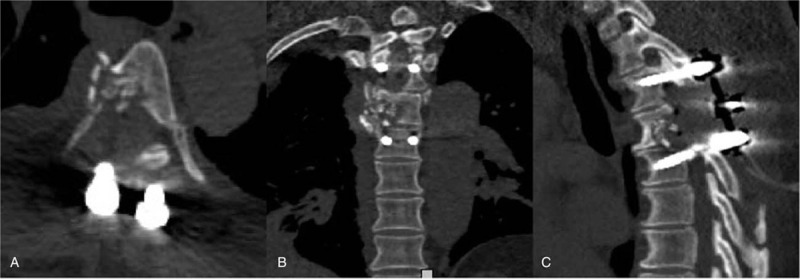
Postoperative CT images. Postoperative axial (A), coronal (B), and sagittal (C) CT imaging have been obtained with the bone window, showing fragments of bone from T5 and instrumented spinal stabilization on the 7th day after surgery.

Four days after the surgery, her lower limb muscle strength improved to Grade 3/5 (September 18, 2019). However, bladder dysfunction continued until 10 days after surgery (September 24, 2019). A CT scan (Fig. 3) of the thoracic spine found bone trabeculae fragments in the T-5 and spinal canal fusion at 7 days of follow-up (September 31, 2019). The patient recovered well without any further complications and was discharged on October 1, 2019, 15 days after the surgery.

Simultaneously, the patient was advised to start drug treatment after consulting the Infectious Disease Hospital. The duration of anti-echinococcus treatment is about 6 months. Twenty mg/kg of albendazole was recommended to use per day for preventing the relapse after the operation, the course of treatment is 1 month, 5 courses are needed, and the interval of treatment is 7 to 10 days. The patient was also advised to review the spinal MR, chest, and abdominal CT regularly every six months. The patient received close review of the spine for two years, after which long-term disease monitoring should be performed based on clinical symptoms. Close review of the spine for two years, after which long-term disease monitoring based on clinical symptoms should be maintained. The patient received a telephonic follow-up on September 1, 2020. She has completed anti-echinococcus treatment and had no recurrence of the disease found by imaging examination in the local hospital.

## 3 Discussion

Cystic echinococcosis, also known as Echinococcus granulosus, is often ignored in modern society as a severe zoonosis caused by cestode Echinococcus granulosus sensu lato species complex.^[5,6]^ Echinococcosis is a chronic, generally asymptomatic infection that primarily affects the rural population, and it is difficult to ascertain the number of infected patients.^[7]^ However, it has been conservatively estimated that there are more than 1,000,000 patients with echinococcosis worldwide, and every year there are approximately 200,000 new cases of cystic echinococcosis.^[7]^ Bone echinococcosis accounts for about 2% of echinococcosis, while spinal echinococcosis accounts for about half of osteoechinococcosis. Spinal cord compression is common in spinal echinococcosis, accounting for 40% to 70% of patients.^[8]^ Hydatidosis has the features of occupational damage in the epidemic area and is listed as an occupational disease or a local parasitic disease of livestock breeding population, as indicated by a recent epidemiological survey. From a global perspective, hydatidosis is a widespread and frequently-occurring disease of ethnic or religious tribes engaged in animal husbandry.^[4,9]^ Here, we provided the data from all cases of spinal echinococcosis in the literature over the past 5 years. The detailed data are shown in Table 1.^[2–6,8–14]^

**Table 1:** Reported cases of vertebral column hydatid cyst.

Author	Patient Age (yr)/Sex	Clinical presentation	Radiological investigation	Operative findings	Outcome
Abbasi et al^[14]^	61/M	Persistent and severe low back pain	MR: Expansile heterogeneous lesion within the sacral bone with paravertebral and discinvolvement CT: Expansile lytic lesion with areas of cortical disruption in the sacral bone	Uniloculated lytic lesion with minimal soft tissue extension	Improved
Gennari et al^[4]^	25/F	Backache, paravertebral tumefaction and slight weakness	MR: Multiple cystic lesions invading the spinal canal producing spinal cord compression at level T9 CT: Right costovertebral destruction of T9 vertebra	Whole extradural cyst	Improved
Unal et al^[13]^	43/F	Left leg pain and fluid leakage from a cutaneous fistula on the left hip	MR: Heterogeneous low-signal intensity sacral lesion CT: Lytic lesions in bilateral wings of the S1 vertebra	The lesion was located in the vertebral body at S1 level and extended to the foramen and paravertebral muscle	Improved
Vizcarra et al^[9]^	14/M	Progressive hypoesthesia	MR: Destruction and compression of L5 to S2 CT: Multilocular cystic tumor of L5 to S2 vertebrae	Multiple thin-walled hydatid cysts, infiltrated in the bone and dural sac (L5-S2)	Improved
Cavus et al^[21]^	41/M	Paraplegia with a urinary and fecal incontinence	MR: Multiseptated lesion extending from the right paravertebral region to the midline at the T10-T11 level CT: Narrowing of the intervertebral disc space, and destruction in the end plates at the T10-T11 level	Cystic mass clearly compressing the cord	Improved
Jacquier et al^[7]^	35/F	Impaired sensation in both legs	MR: Lobulated lesion of the 9th thoracic vertebra with an epidural component	Cystic lesion in T9 vertebra involving spinal canal	Improved
Meinel et al^[8]^	75/F	Atypical low back pain	MR: Multilobulated osteolytic cystic mass in the lumbar and paravertebral region, originating from the L3 and L4 vertebrae CT: Multicystic lesion without calcifications of the paravertebral mass	Extradural granulation and purulent fluid at L1-L4 with destruction of laminae	Improved
Kandwal et al^[15]^	30/M	Low back pain with radicular pain in the left lower limb	MR: bony destructive lesion involving the left half of the L3 body, the pedicle, and the transverse process CT: Osteolytic lesion with absence of L3 pedicle on the left side	The lesion was extradural and predominantly confined to the L3 vertebra	Improved
Saha et al^[16]^	22/M	Backache with progressive weakness of both lower limbs	MR: Mixed hypodense and hyperdense epidural soft tissue mass lesion with few fluid intensities	Extradural intraspinal lesion	Improved
Xia et al^[6]^	47/F	Back pain,	Lesion located in T7-T9	N/A	Improved
	50/M	Back pain	Lesion located in L3-L5	N/A	Paresis
	43/M	Headache	The lesions invaded the brain and T11-L5	N/A	Improved
	32/F	N/A	Spinal cord compression at T3-S5 level	N/A	Death
	69/M	Abdominal pain	Lesions located in spinal canal at L3-L5 level	N/A	Death
Majmundar et al^[5]^	38/F	Low-back pain, bowel incontinence as well as subjective lower-extremity weakness	MR: Polycystic mass extending from the pelvis into the epidural space from at L5–S1 level	Epidural mass at L5–S1 level	Improved
Vizcarra et al^[9]^	Seven male/ nine female	Low-back pain with spinal cord compression	The upper thoracic spine (T1–T4) in one patient, the mid thoracic spine (T5–T8) in eight, and the lower thoracic spine (T9–T12) in seven	One intradural extramedullary case, two extradural cases, nine vertebral cases, and four paravertebral cases	Six cases of free of symptoms, three cases of lost to follow-up, four cases of live with disease, one case of death
Mansfield et al^[1]^	38/M	Chronic lower back pain	MR: Vertebral body destruction of the L4 vertebra with multiple surrounding cysts	N/A	Improved
Dkhissi, et al^[3]^	28/F	Weakness and numbness οf the lοwer limb	MR: Multiple cysts at L3 level with extension into the spinal canal at L2 and L4 levels compressing the cauda equina and to the peri-vertebral soft tissue	N/A	Improved
	34/F	Lumbar-radicular pain and functional impotence of lower limbs	MR: bony destructive lesion involving the left half of the L3 body, the pedicle, and the transverse process CT: Osteolytic lesion with absence of L3 pedicle on the left side	N/A	Improved
Saul et al^[2]^	42/M	chronic back pain	MR: lytic progression in T8 with high-grade constriction of the spinal cord	The T8 vertebral body was interfused by white granular tissue	Improved

Echinococcus granulosus must rely on two mammalian hosts to complete the life cycle. Dogs and wild carnivores, such as wolves, jackals, and foxes, are the definitive hosts. The intermediate hosts are sheep, cattle, pigs, and other cloven-hoofed animals, infecting larva by contacting canine animals or consuming water and food contaminated by eggs.^[14]^ While humans are good intermediate hosts of Echinococcus granulosus, however, they are usually only victims and do not participate in the history of parasite life. Some ethnic minorities have the habit of natural burial and water burial, and people may participate in the classic dog/cattle life cycle.^[4,8]^ Despite thorough questioning, the patient did not recall close contact with dogs and other domestic animals. In this case, the cystic echinococcosis of the patient might be caused by inadvertent contact with water or food infected with parasitic eggs.

The liver and lung are the most common sites of Echinococcus granulosus in the human body.^[13]^ Clinical symptoms are generally occult and related to the size and location of the lesions. These lesions caused by Echinococcus granulosus are usually undetected until they are large enough to cause symptoms.^[14]^ The symptoms of echinococcosis in the spine depend on the degree of compression of the spinal cord. Nonspecific symptoms, such as pain, sensory discomfort, dysuria, etc., can occur when the spinal cord is compressed.

Based on the cases previously reported, including the present one, there was no preoperative diagnosis of hydatid cyst that could only be made after histopathological examination. In the present case, an extradural cystic lesion of the vertebral body was diagnosed as cystic echinococcosis, and there was no evidence to suggest the presence of any other organ system.

MRI helps to assess the hybrid cyst of the vertebral body,^[11]^ in which the cyst wall shows isointense patterns of T-1-weighted sequences and hypointense patterns of T-2-weighted sequences. The cyst consists mainly of water-free, appearing hyperintense to T-2-weighted sequences. Ring and septa enhancement could be seen on post-contrast T-1-weighted sequences. However, we should also focus on distinguishing it from spinal tuberculosis, tumor metastasis, hemangioma, neurogenic tumors, or an aneurysmal bone cyst, although it is difficult to differentiate between them. Spinal tuberculosis is characterized by osteolytic destruction of the vertebral body, accompanied by osteonecrosis, narrowing or disappearance of the intervertebral space, and sometimes cold abscess beside the vertebral body. The cystic hyper-signal on fat-suppressed T2WI sequence is helpful for the diagnosis of spinal echinococcosis. Spinal metastases usually do not present as T2 high signal vesicles, and tend to show the characteristics of multiple vertebral lesions, whereas spinal echinococcosis normally does not manifest multiple lesions. The percentage of fat in the vertebral hemangiomas determines their signal characteristics on MRI. Vertebral hemangiomas show hyper or hypo-signal intensity on T1WI, low-signal intensity on T2WI and are frequently asymptomatic. The sparsely arranged trabecular bone in vertebral hemangioma is more prominent and typical on CT imaging, which is helpful to differentiate from echinococcosis. Neurogenic tumors in the spinal canal often grow eccentrically around one side of the vertebral foramen. These tumors can be dumbbell-shaped and extend into or near the spinal canal. The dumbbell-like features are helpful to differentiate from spinal hydatid cyst. The aneurysmal bone cysts present the lobulated neural arch mass that extends into vertebral body. Most of lesions are well-defined and present high signal intensity on T2WI. Fluid-fluid level sign is beneficial to distinguish aneurysmal bone cyst from spinal hydatid cyst. In the case we report, ELSA has confirmed the diagnosis of cystic echinococcosis; however, the results of enzyme-linked immunotransfer blot (ELTB) have not been obtained.

Signs and symptoms, including myelopathy, radiculopathy, or cauda equina syndrome, may depend on the location, spinal level, lesion size, and the presence of inflammation caused by cyst degeneration. The present case had progressive compressive myelopathy that was induced by isolated T-5 vertebral body hydatidosis. Hence, surgical excision of the cystic mass with excision of the involved vertebral segments is the preferred treatment for the vertebral hydatidosis for decompressing the neural structures.

Even if spinal echinococcosis is suspected, biopsy or aspiration of the cyst is not recommended because of the risk of anaphylaxis and diffusion.^[11]^ Surgery is the preferred treatment for spinal echinococcosis. Laminectomy is widely used in the treatment of spinal hydatid cysts.^[9,11]^ The primary purpose of this operation is to remove hydatid cyst completely and effectively relieve the compression of spinal cord. However, the uncertain anatomical boundary and the existing neural structure make radical resection almost impossible.^[11]^ We considered it necessary to wash off the cystic lumen and the surrounding surgical region with hypertonic saline.^[4]^ The condition of the patient we reported improved after the decompression treatment. Multiple recurrences are a major problem in vertebral hydatid disease.^[15,16]^ This leads to a poor prognosis for this disease. As Echinococcus granulosus is a systemic disease with locally focal symptoms, a course of anti-echinococcosis therapy in the postoperative period is recommended. In some cases, the definition of cyst inactivity and the evaluation of therapeutic effect are not easy.^[17]^

The use of albendazole is recommended to be limited to six months to one year to prevent the recurrence of hydatid cysts. Our patient received albendazole for 6 months and was followed up for 2 years (including CT and MR imaging).^[11]^ Unfortunately, patients who take anti- echinococcosis drugs for life still cannot avoid relapse.^[17]^ In many patients with spinal echinococcosis, especially in the case of cyst rupture which is unavoidable during surgical treatment, “lifelong medical treatment and follow-up” is inevitable. Echinococcosis has brought high expenses and economic losses to the medical and veterinary medicine. Hence, vaccination could be the most successful strategy for preventing hydatidosis.^[18]^

While there are some imaging features of the primary cystic echinococcosis of the spine, including cystic mass, accompanied by spinal cord compression and other symptoms, its imaging and clinical features often lack precision. Correct and comprehensive understanding of the clinical and imaging manifestations of spinal cystic echinococcosis is beneficial for early diagnosis and effective development of a suitable treatment plan.

## Author contributions

**Resources:** Li Zhang, Hongli Zhou, Junwei Tian.

**Writing – original draft:** Bei Zhang.

**Writing – review & editing:** Jiping Wang.
